# Improvement Properties of Hybrid Halide Perovskite Thin Films Prepared by Sequential Evaporation for Planar Solar Cells

**DOI:** 10.3390/ma12091394

**Published:** 2019-04-29

**Authors:** Miguel Á. Reinoso, Camilo A. Otálora, Gerardo Gordillo

**Affiliations:** 1 Departamento de Física, Universidad Nacional de Colombia, 111321 Bogotá, Colombia; ggordillog@unal.edu.co; 2 Facultad de Ciencias de la Ingeniería, Universidad Estatal de Milagro, 091706 Milagro, Ecuador; 3 Departamento de Química, Universidad Nacional de Colombia, 111321 Bogotá, Colombia; caotalorab@unal.edu.co; 4 Académicos por Colombia, 111321 Bogotá, Colombia

**Keywords:** Perovskite thin films, MAPI, FAPI, microstructure, planar perovskite solar cells

## Abstract

Thin films of CH_3_NH_3_PbI_3_ and (NH_2_)_2_CHPbI_3_ (from now on abbreviated as MAPI and FAPI respectively), with perovskite structure were prepared by sequential evaporation of lead iodide (PbI_2_) and methylammonium iodide (MAI) or formamidinium iodide (FAI), with special emphasis on the optimization of its optical, morphologic, and structural properties. For this, the evaporation process was automatically controlled with a system developed using virtual instrumentation (VI) that allows electronic control of both evaporation sources temperature and precursors deposition rates, using proportional integral derivative (PID) and pulse width modulation (PWM) control algorithms developed with the LabView software. Using X-ray diffraction (XRD), information was obtained regarding the phase and crystalline structure of the studied samples as well as the effect of the main deposition parameters on crystallite size and microstrain. We also studied the influence of the main deposition parameters on the optical and morphological properties through measurements of spectral transmittance and scanning electron microscopy (SEM) respectively. It was found that the implemented method of sequential evaporation allows preparing, with a high degree of reproducibility, single phase MAPI and FAPI thin films with appropriate properties to be used as active layer in hybrid solar cells. The applicability of MAPI and FAPI thin films as active layer in photovoltaic devices has been demonstrated by using them in solar cells with structure: FTO/ZnO/MAPI(or FAPI)/P3HT/Au.

## 1. Introduction

Hybrid perovskites are a family of organic-inorganic materials [1,2] of interest for several applications including solar cells, optoelectronic [3], and colloidal semiconductor nanocrystals (NCs) [4]. Solar cell technology based on organic-inorganic hybrids perovskites, have had significant progress in only few years [5,6,7,8,9]. In a short period of time, efficiencies greater than 22% [10] have been achieved, being 23.7% the current record of efficiency [11]. Currently the solar cells of perovskites are emerging as possible candidates to make the transition to an industrial level production [7]. However, to fully understand their function, it is necessary to investigate aspects related to the mechanisms that affect their properties. The search for alternatives to improve their stability, moisture sensitivity, or rapid degradation when exposed to oxygen and light—either materials or devices—is also a very important issue [12,13,14].

In general, the performance of photovoltaic devices based on hybrid perovskites is affected by the thin film deposition technique, thickness, and composition. The vacuum evaporation technique offers the possibility of depositing thin films of hybrid perovskites, without the limitations characteristic of deposition in solution based mainly in spin-coating. On the other hand, evaporation process from two separated crucibles allows precise control of MAPI and FAPI films stoichiometry; the evaporation technique also allows deposition of high purity layers with good homogeneity in thickness and composition [5,15]. The evaporation of hybrid organic–inorganic compounds using a single source presents difficulties because the organic precursor generally degrades at temperatures lower than the evaporation temperature of the inorganic compound; therefore, it is more convenient to deposit these compounds using two separate evaporation sources, one for the organic precursor and other for the inorganic.

The main motivation of this work was to optimize a synthesis route based on the sequential evaporation of precursors to grow, with a high degree of reproducibility, thin films of MAPI and FAPI with suitable properties for solar cells. A specially designed equipment was implemented to achieve this purpose, equipment includes an electronic system that allows precise control of both evaporation sources temperature and precursors deposition rates, using PID and PWM control algorithms developed with LabView software. For the fulfillment of this objective a study was conducted to optimize the optical, structural, and morphological properties of thin films of MAPI and FAPI; this was achieved through a correlation of synthesis parameters with characterization results made through transmittance, XRD and SEM. Efficiencies around 9.4% were achieved with solar cells fabricated with structure FTO/ZnO/MAPI/P3HT/Au.

## 2. Experimental

The growth of perovskite films was done by sequential evaporation of precursors (PbI_2_ and MAI or FAI) following a procedure in two stages. In a first stage the crucibles were heated at a constant temperature ramp to a reference temperature controlled through a PID algorithm. Once the crucible temperature reached the evaporation temperature of the respective precursors, the PID controller was disabled and the heating of the crucibles was controlled based on the deposition rate by a PWM algorithm using a thickness monitor as sensor. 

Generally, the deposition of this type of compound is done by evaporating the precursors, controlling only the evaporation temperature of these; however, we decided to use a two-stage process because this way it was possible to significantly improve the properties and the reproducibility of the samples.

**Figure 1:**
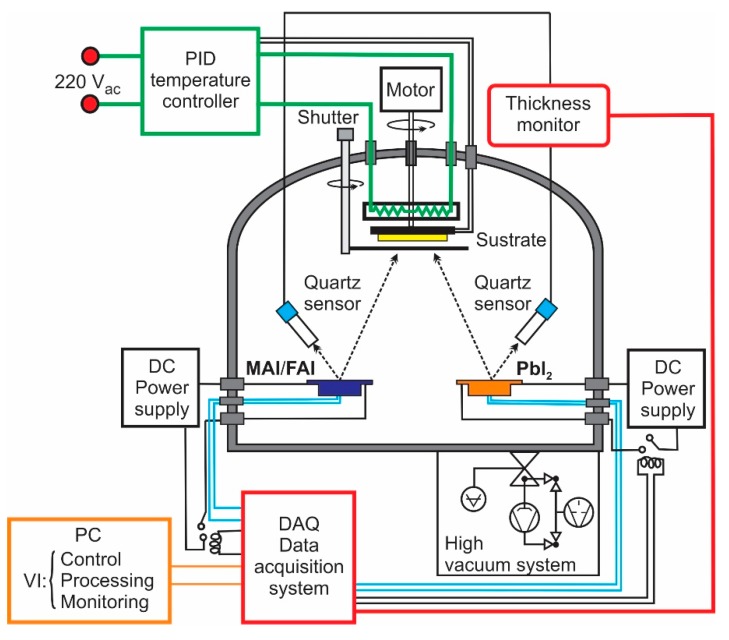
Scheme of the system used to grow CH_3_NH_3_PbI_3_ (MAPI) or (NH_2_)_2_CHPbI_3_ (FAPI) films by sequential evaporation of precursors. PID: proportional integral derivative control algorithm.

Figure 1 shows a scheme of the equipment we have implemented to grow MAPI and FAPI thin films following a route that includes sequential evaporation of their precursors. This includes the following units:

1. High vacuum system, which allows to achieve a basis pressures around 2 × 10^−5^ mbar
2. Rotating substrate heating unit with facilities to control the temperature through a commercial PID system
3. Knudsen cell-type evaporation sources heated through a thermocoax heating element.
4. Unit for automatic control of the evaporation process, constituted by a system of acquisition and processing of data and a virtual instrument to control both the crucibles temperatures and the deposition rate of the precursors. The control of the temperature of evaporation sources and the deposition rate are achieved through PID and PWM algorithms respectively. This unit also has facilities to monitor in real time the evolution of the temperature and the deposition rate of the precursors, as well as the thickness of the samples.
5. Maxtek thickness monitor TM-400 (MaxTek Inc., Cypress, CA, USA) that allows measuring both the deposition rate and the thickness of PbI_2_, MAI and FAI layers. The real thickness ($\delta_{real}$) of perovskite samples is determined by multiplying the thickness measured by the monitor ($\delta_{monit}$) by a factor K established by means of comparison between the thickness measured by the monitor and the measured one using a Veeco Dektak 150 surface profiler (Veeco Instruments Inc., Plainview, New York, USA) ($\delta_{profiler}$) that is to say $K=\delta_{profiler}/\delta_{monit}$.

MAPI and FAPI films were deposited evaporating sequentially PbI_2_ (commercial reagent Sigma Aldrich, St. Louis, MO, USA) and MAI (FAI) synthesized in our laboratory following the procedure described in Ref. [16].

The samples were grown using the following routine:

1. Initially a constant temperature ramp (around 15 °C/min) is established for the crucible of PbI_2_ until reaching the temperature at which evaporation begins (340 °C); subsequently, the deposition of the PbI_2_ layer starts at a constant deposition rate. The deposition of PbI_2_ finishes when the wished thickness has been reached.
2. In a second stage, on the PbI_2_ layer, MAI (or FAI) is evaporated following a similar procedure to that used for the PbI_2_. MAI and FAI layers were evaporated at 260 °C and 180 °C respectively
3. After the deposition the samples are annealed in nitrogen atmosphere for 20 min at temperatures between 100 and 140 °C.

It is worth mentioning that the electronic facilities included in the evaporation system we have designed and implemented for the deposition of perovskites, allow to control with precision and high degree of reproducibility the stoichiometry and crystallographic phase of the samples. These properties are typically required to fabricate high-performance photovoltaic (PV) devices. This type of electronic control is not included in commercially available thermal evaporation systems.

For designing an evaporation route, it is important to taking into account the evaporation, sublimation or degradation process which can occur during the heating of the chosen precursors. In this particular case of MAPI and FAPI synthesis has been found that FAI decomposes at temperatures above 252 °C [17], MAI sublimation occurs at a temperature of above 240 °C without degradation [18] and PbI_2_ thermally decomposes if the temperature is higher than 646 °C; it means that the evaporation temperatures used in this paper does not induce degradation of the precursors.

The conditions to grow MAPI and FAPI films with perovskite structure and improved properties were found through a parameter study which was conducted to determine the effect of the main deposition parameters on the phase as well as on the optical, electrical, structural and morphological properties. Table 1 lists the parameters studied and its range of variation.

It is worth mentioning that the calculation of the molar ratio of precursors from the knowledge of the ratio of thicknesses is very simple to do, by multiplying the volume of the sample (cross section area of sample multiplied by the thickness) by its density to obtain the mass; the mass can finally be converted into the molar amount by dividing it into the molar mass. For example, the thickness ratio of 2.6:1 in the case of PbI_2_/MAI correspond to a molar ration approximately equal to 1:1.

We decided to study a wide range of compositions because we were interested in studying the effect of excess of PbI_2_ and of MAI on both the composition and the properties of MAPI films. In addition, we consider it advisable to study a wide range of compositions because in evaporation techniques, the molar ratio used in the crucibles is not necessary the molar ratio obtained in the substrate. It is also important to mention that this work was the first approximation of perovskite synthesis by evaporation using this machine and it is interesting to evaluate the response of the implemented deposition system under a wide range of synthesis parameters.

The samples prepared were characterized by means of transmittance and reflectance measurements performed using a Varian–Cary 5000 spectrophotometer (Agilent Technologies, Santa Clara, CA, USA), as well as by XRD measurements performed with a Philips X’Pert Pro PANalytical diffractometer (Malvern Panalytical Ltd., Malvern, UK), using the radiation Cu-Kα (1.540598 Å), an acceleration voltage of 40 KV and a current 40 mA. SEM images were performed with a TESCAN Vega 3 scanning electron microscope (Tescan, Brno, Czech Republic).

## 3. Results and Discussion

### 3.1. Structural Characterization

**Figure 2:**
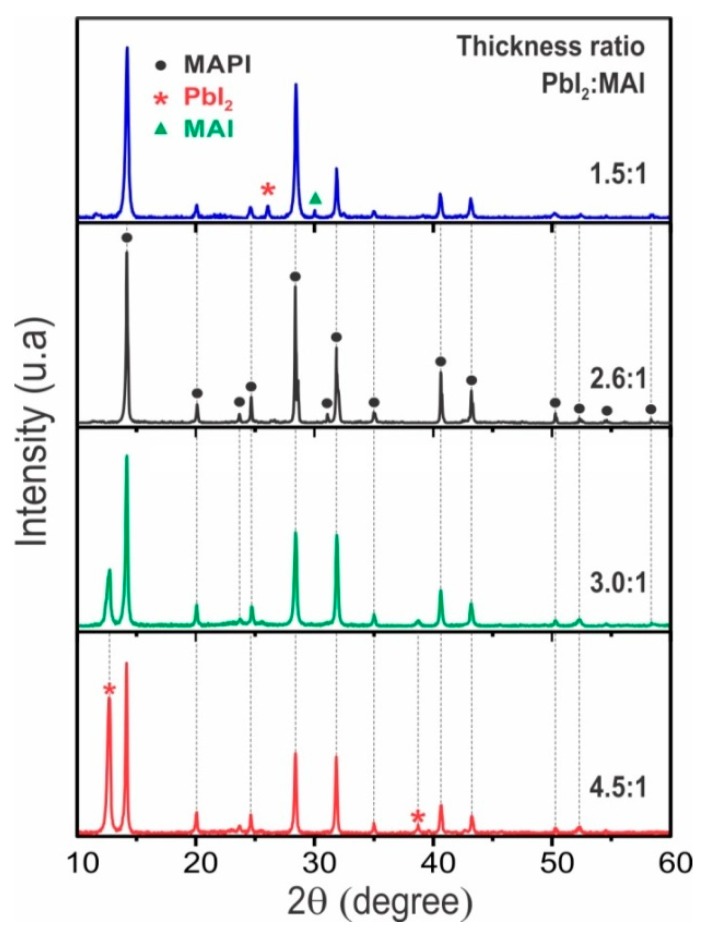
X-ray diffraction spectra of MAPI films prepared varying the lead iodide (PbI_2_)/MAI thickness ratio.

The influence of chemical composition (determined from the thickness ration PbI_2_/MAI or PbI_2_/FAI) and post deposition annealing on the phase, structure and microstructure of MAPI thin films grown by sequential evaporation were determined through XRD measurements. XRD spectra of MAPI films prepared varying the chemical composition, are displayed in Figure 2. The diffractograms exhibit reflections which have been assigned to MAPI with perovskite structure [19,20,21,22,23] but also reflections associated to precursor materials such as the peaks in 2θ = 12.6° and 2θ = 26.3° that have been assigned to the PbI_2_ hexagonal phase [24,25] and 2θ = 31.7° assigned to the MAI phase [26]; these results indicate that samples deposited with excess of MAI (ratio 1.5:1) present a mixture of the phases MAPI, MAI, and PbI_2_, while those with excess of PbI_2_ (ratio 4.5:1 and 3:1) grow with a mixture of the phases MAPI and PbI_2_. However, we were able to grow samples of MAPI free of secondary phases using a PbI_2_/MAI thickness ratio close to 2.6:1.

**Figure 3:**
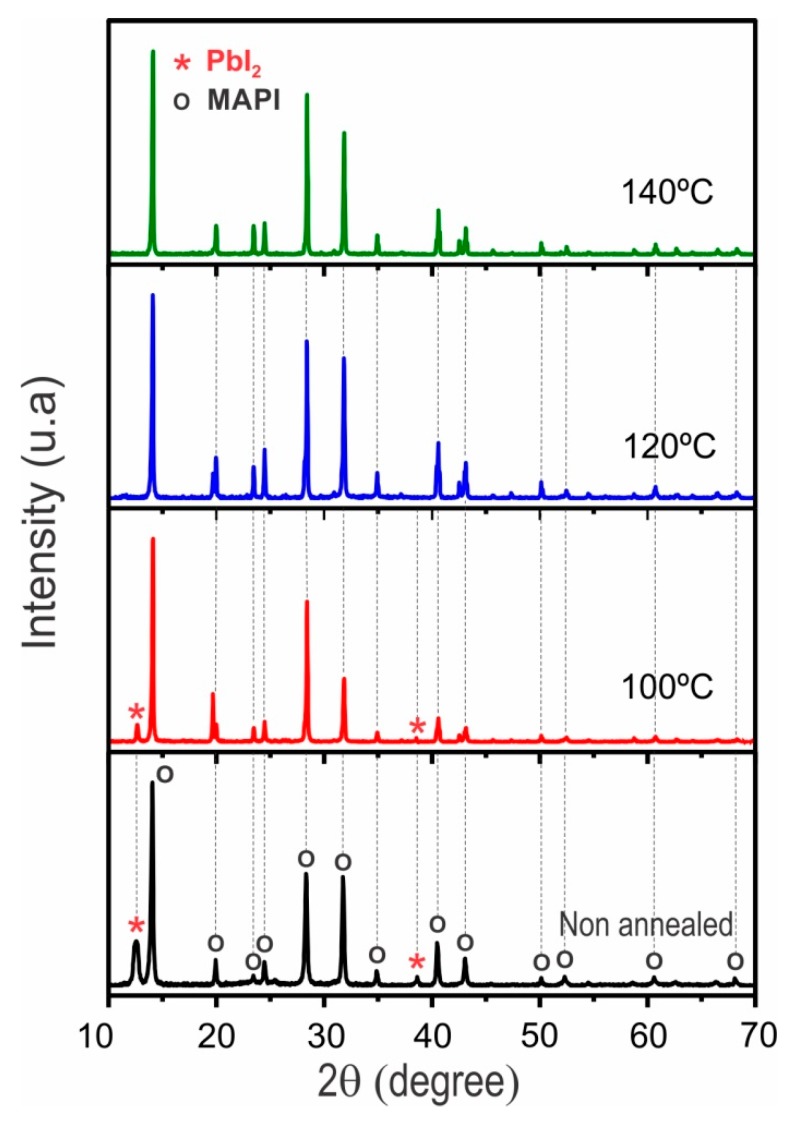
X-ray diffraction (XRD) spectra of a MAPI film prepared under a PbI_2_/MAI thickness ratio of 3:1, followed by post deposition annealing in N_2_-atmosphere for 20 min at temperatures varying between 20 and 140 °C.

Figure 3 shows the effect of post deposition annealing during 20 min at different temperatures on the XRD pattern of a MAPI film prepared under a PbI_2_/MAI thickness ratio of 3:1. It is observed in Figure 2 that this type of samples grows mainly in the MAPI phase, however they also contain a small amount of PbI_2_. The PbI_2_ secondary phase can be reduced or eliminated by post deposition annealing in atmosphere of nitrogen. The results (Figure 3) indicate that annealing for 20 min at 100 °C reduce the PbI_2_ amount while annealing at higher temperatures leads to PbI_2_ free perovskite samples.

**Figure 4:**
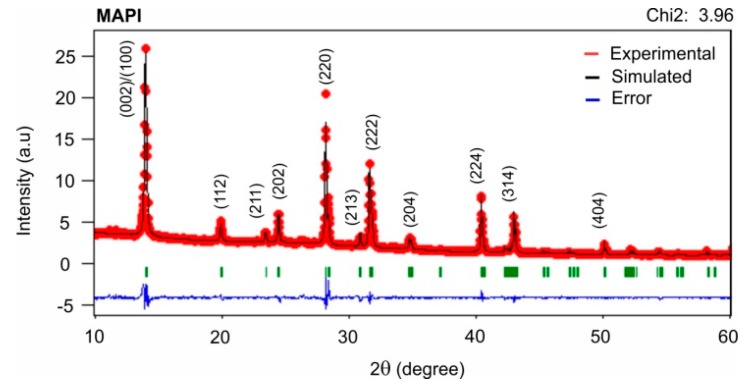
Simulated and experimental difractograms of MAPI film (PbI_2_/MAI thickness ratio of 2.6:1).

The crystalline structure of a typical MAPI sample prepared under a PbI_2_/MAI thickness ratio of 2.6:1, as well as the indices of Miller associated to the identified reflections were determined by comparing the experimental XRD spectrum with a theoretically simulated difractogram, using the FullProf program; the simulation was done assuming samples with known crystalline structure and group of symmetry and additionally using crystallographic parameters reported in the literature [19,21,22]. The simulation performed fitted quite well to the experimental results (Figure 4 with chi-squares less than 4). From these results was established that this type of MAPI films grows with tetragonal structure and I4cm symmetry group.

**Figure 5:**
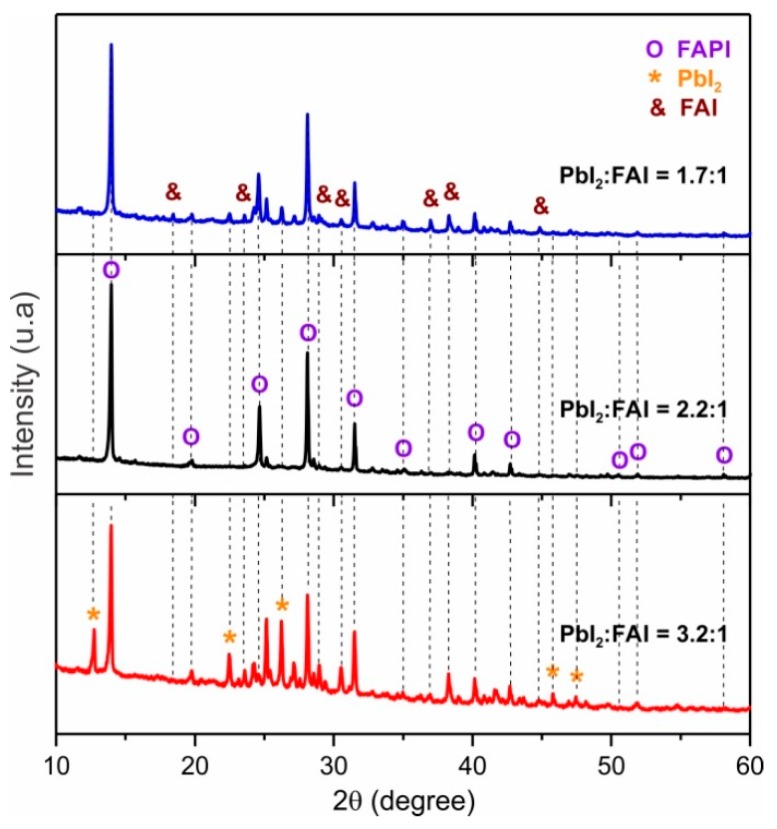
XRD spectra of FAPI film prepared varying the PbI_2_/FAI thickness ratio.

Figure 5 shows XRD spectra of FAPI films prepared varying the chemical composition. The diffractograms exhibit reflections in 2θ = 13.93°, 28.07° and 31.47°, which correspond to FAPI trigonal phase [27,28,29] but also shown reflections associated to other phases, depending on the PbI_2_/FAI deposition rate ratio. Samples with excess of FAI (PbI_2_/FAI thickness ratio of 1.7:1) exhibits peaks of the tetragonal FAPI phase, but also other reflection in 2θ = 28.9°, 38.2°, 44.9° from monoclinic FAI phase [17] and in 2θ = 22.3° y 26.2° from PbI_2_ trigonal phase [24]. The XRD pattern of Figure 5 also show that the samples deposited with excess of PbI_2_ (PbI_2_/FAI thickness ratio of 3.2:1) grow with a mixture of the phases FAPI, FAI, and PbI_2_. However, it was found that it is possible to obtain samples of FAPI free of secondary phases using a PbI_2_/FAI thickness ratio close to 2.2:1, without having to perform a post-annealing.

**Figure 6:**
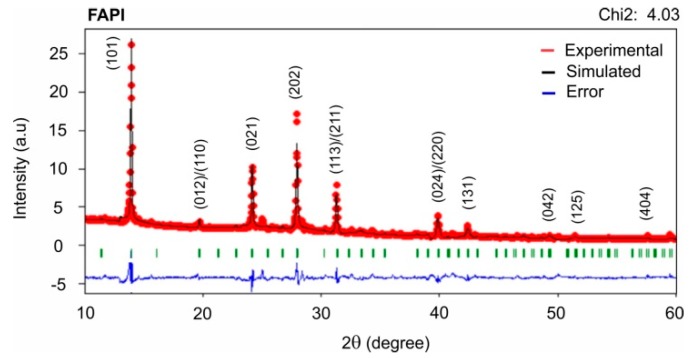
Simulated and experimental XRD patterns of a typical FAPI film (PbI_2_/FAI thickness ratio of 2.2:1).

The crystalline structure of a typical FAPI film prepared under a PbI_2_/FAI thickness ratio of 2.2:1 was determined by comparing the experimental XRD spectrum with a theoretically simulated difractogram; the simulation was performed assuming samples with known crystalline structure and symmetry group, and additionally using crystallographic parameters reported by other authors [29,30]. The simulated XRD spectrum fitted quite well to the experimental (see Figure 6). From these results was established that this type of FAPI films grow with trigonal structure and P3m1 symmetry group.

In general, the full width at half maximum (FWHM) value of the MAPI and FAPI films is affected by both the composition and the annealing temperature, indicating that these parameters affect its crystallinity. Considering that the peak broadening is affected by crystallite size D and lattice strain *ε* (relative change in size with respect to the size before experiencing an external force) induced by structural defects including dislocations, vacancies, stacking faults interstitials, among others [31,32], these two parameters can be determined by the X-ray line broadening method using the Williamson–Hall equations [33] given by:

(1) $$\beta_{hkl}cos\theta_{hkl}=\frac{K\lambda }{D}+4\;\varepsilon sin\theta_{hkl}$$

where D is the crystallite size, λ is the wavelength of the CuKα radiation, k is a constant equal to 0.94, $\beta_{hkl}$ is the peak width at half-maximum intensity and $\theta_{hkl}$ is the peak position. 

Since the XRD peak broadening is affected by instrumental effects, in this work the instrumental corrected broadening $\beta_{hkl}$ was estimated using the following equation [34]:

(2) $$\beta_{hkl}={\left[{\left(\beta_{hkl}\right)}_{measured}^{2}-\;{\left(\beta_{hkl}\right)}_{instrumental}^{2}\right]}^{1/2}$$

where the instrumental broadening $\beta_{instrumental}$ was determined using the Cagliotti relationship [35] and the parameters U, V and W of this relation were determined by fitting the XRD spectrum of a standard of Si with a spectrum simulated theoretically using the FullProf program. 

Assuming that the strain is uniform in all crystallographic directions, Equation (1) represents the UDM model (Uniform Deformation model). Plotting the term βcosθ with respect to 4sinθ for the preferred orientation peaks of MAPI and FAPI films, the strain and crystallite size can be obtained from the slope and y-intersect of the fitted line. 

The values of D and Ɛ estimated from the UDM model and average values of grain size determined through SEM images (with the help of the Image-J program), are summarized in Table 2. The crystallite size D and parameter Ɛ of MAPI and FAPI samples deposited under conditions different to those reported in Table 2, were not calculated because this type of samples have secondary phases whose diffraction peaks overlap to those of the MAPI phase, which does not allow to determine with precision the $\beta_{hkl}$ values. 

The results of Table 2 reveal that the MAPI films deposited grow with similar grain size but larger than the sample of FAPI. It is also observed that the MAPI samples prepared from a thickness ratio of 2.6:1 exhibit lower values of Ɛ, indicating that these grow with a lower density of structural defects as compared with the other two types of studied samples.

### 3.2. Optical Properties

**Figure 7:**
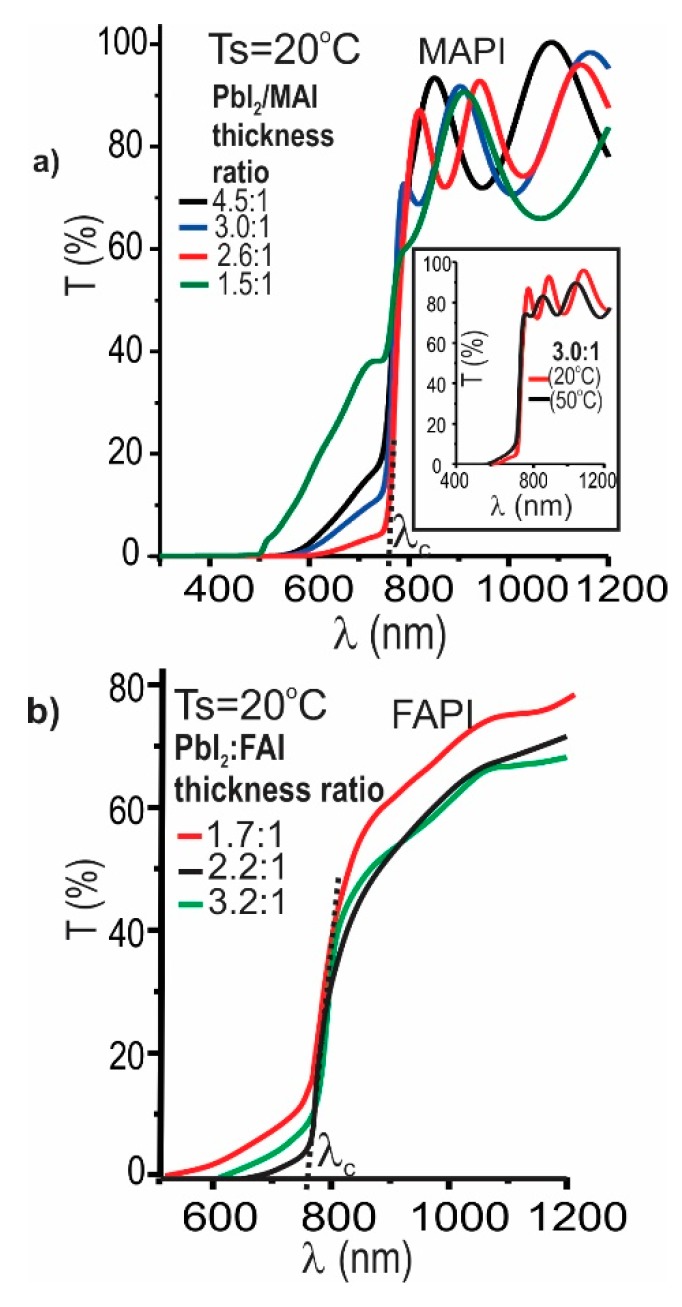
Influence of the PbI_2_/MAI and PbI_2_/FAI thickness ratio on the transmittance spectra of (**a**) MAPI and (**b**) FAPI films prepared at room temperature; in inset are plotted transmittance curves of MAPI films prepared under a thickness ratio of 3:1 and deposited varying the substrate temperature between 20 and 50 °C.

The influence of chemical composition and post deposition annealing on the optical properties of MAPI films was studied through spectral transmittance and reflectance measurements. Figure 7a shows the transmittance spectra of MAPI films prepared at room temperature, varying the PbI_2_/MAI thickness ratio; the inset show transmittance curves of MAPI films prepared under a thickness ratio of 3:1 and deposited varying the substrate temperature between 20 and 50 °C. In Figure 7b are also shown transmittance spectra of FAPI films prepared at room temperature, varying the PbI_2_/FAI thickness ratio.

The transmittance curves shown in Figure 7 reveal that at wavelengths greater than the cutoff wavelength (λc), the substrate temperature and the relation PbI_2_/MAI (or PbI_2_/FAI) do not affect neither the intensity nor the slope of the transmittance curves; nevertheless, these parameters affect the shoulder observed at λ< λc, that apparently is caused by the presence of secondary phases.

It is also observed in Figure 7 that the shoulder becomes more pronounced in both samples rich in PbI_2_ as in samples rich in MAI (or FAI). Correlating the measurements of transmittance with those of XRD shown in Figure 2 and Figure 5, we can conclude that the appearance of the shoulder is given as a consequence of the formation of secondary phases of PbI_2_, MAI, and FAI. On the other hand, the inset of Figure 7a shows that the increase of substrate temperature results in an increase in the shoulder size, associated with excess PbI_2_ induced by the re-evaporation of the organic precursor (MAI).

**Figure 8:**
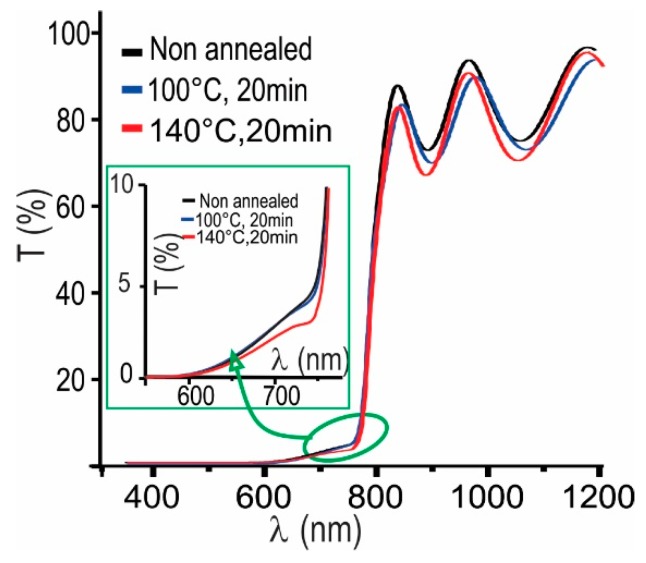
Effect of annealing temperature on the transmittance of a typical MAPI film prepared under a PbI_2_/MAI thickness ratio of 3:1.

Figure 8 shows the effect of the annealing temperature on the spectral transmittance of MAPI films prepared under a PbI_2_/MAI thickness ratio of 3:1.

From this result can be seen that the transmittance at wavelengths lower than λ_c_ of MAPI films prepared with excess of PbI_2_, decreases significantly when these are annealed at temperatures of 140 °C, indicating that this type of annealing prevents the secondary phase formation. This result agrees with those obtained through XRD measurements (Figure 3).

Considering that MAPI and FAPI samples deposited at room temperature under PbI_2_/MAI ratio of 2.6:1 and PbI_2_/FAI ratio of 2.2:1 respectively do not present secondary phases; these samples were selected to determine the absorption coefficient α and the energy gap (Eg) using the Equations (3) and (4) respectively [36].

(3) $$\alpha =-\frac{1}{d}\left[ln\left(\frac{T(\lambda )}{1-R(\lambda )}\right)\right]$$

(4) $${\left(\alpha h\nu \right)}^{2}=A_{n}\;\left(h\nu -E_{g}\right)$$

where *d* is the film thickness, *T* is the transmittance and *R* the reflectance. *Eg* value was obtained from the intercept with the axis hν of the curve (αhν)^2^ versus hν.

**Figure 9:**
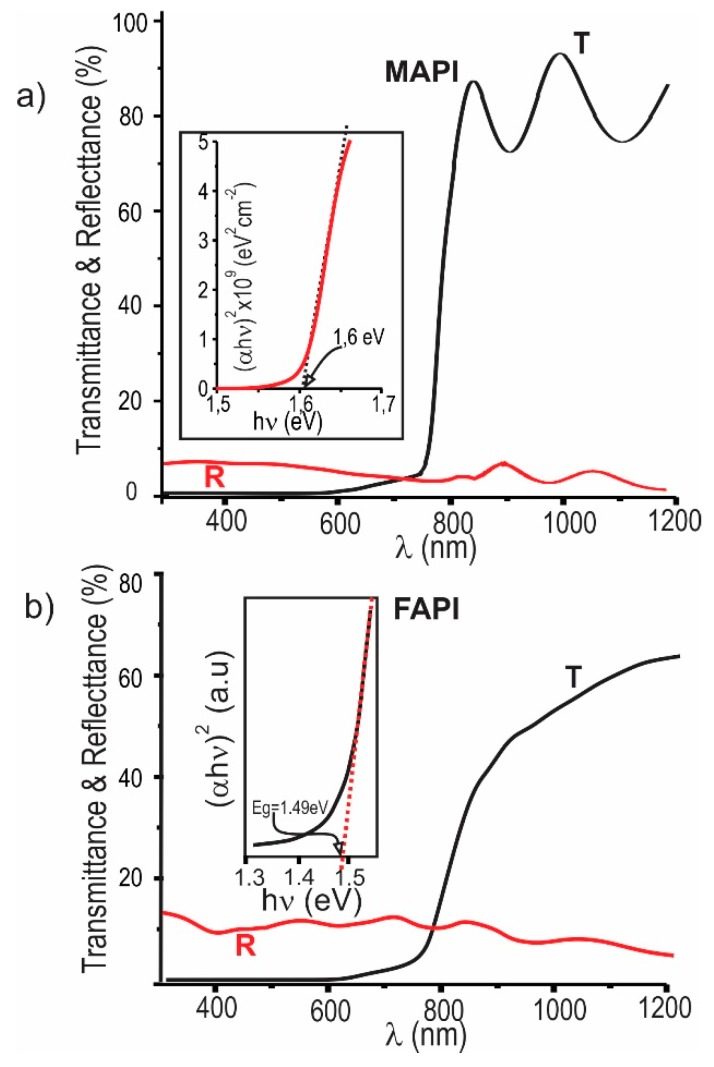
Transmittance and reflectance spectra of typical samples of (**a**) MAPI and (**b**) FAPI films free of secondary phases; in inset are displayed curves of α versus λ and of (αhν)^2^ versus hν.

Figure 9 shows transmittance and reflectance spectra corresponding to typical MAPI and FAPI samples free of secondary phases and in the inset are displayed curves of (αhν)^2^ versus hν used to determine the Eg value. It was found that MAPI and FAPI films have energy gaps of 1.60 and 1.49 eV respectively, these agrees with Eg values reported by other authors [37].

### 3.3. Morphological Characterization

**Figure 10:**
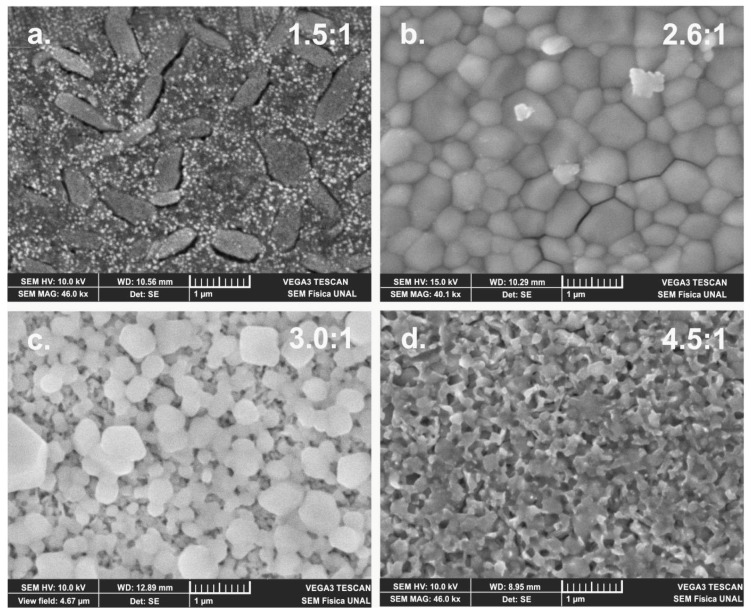
SEM images of MAPI films prepared at room temperature and PbI_2_/MAI thickness ratio of (**a**) 1.5:1, (**b**) 2.6:1, (**c**) 3.0:1 and (**d**) 4.5:1.

The influence of chemical composition and post deposition annealing temperature on the morphology of MAPI and FAPI films was studied through SEM measurements. Figure 10 shows SEM images corresponding to MAPI films prepared at room temperature varying the PbI_2_/MAI thickness ratio. This study reveals that the morphology of the MAPI films is affected by both, the chemical composition and annealing temperature. In particular, it is observed that the samples prepared with excess of MAI (1.5 to 1 ratio) present formation of large and elongated clusters (≈ 0.3 × 0.8 µm) surrounded by grains of nanometric size. Taking into account the results of XRD that indicate that samples deposited with excess of MAI present formation of the MAI phase, we believe that the observed clusters could correspond mainly to the compound MAI. On the other hand, samples prepared with excess of PbI_2_ (4.5 to 1 ratio) exhibit a porous morphology constituted by grains of sub-micron size of irregular shape. The SEM images also show that the MAPI samples prepared with chemical composition corresponding to a PbI_2_/MAI thickness ratio of 2.6:1 exhibit a morphology consisting of compact grain structures with sub-micron size; it was found that the grain size of this type of MAPI samples vary between 0.3 and 0.8 µm, with an average grain size of around 0.49 µm.

**Figure 11:**
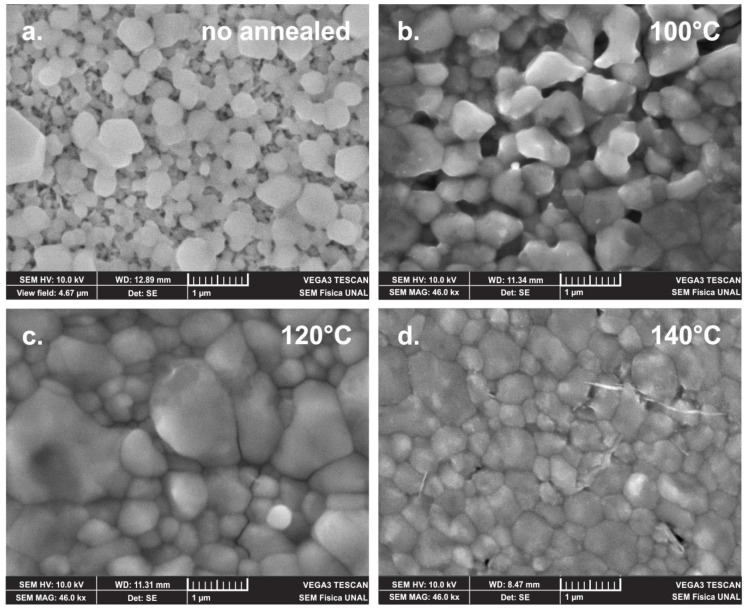
SEM images of MAPI films with composition corresponding to a PbI_2_/MAI thickness ratio of 3:1, (**a**) no annealed, and at annealed temperatures of (**b**) 100 °C, (**c**) 120 °C and (**d**) 140 °C.

In Figure 11 are displayed SEM images of a MAPI film prepared at room temperature under a PbI_2_/MAI thickness ratio of 3:1 and annealed after deposition at temperatures ranging between 100 and 140 °C. These results show that post-annealing treatment induces significant changes in the morphology of the MAPI films, among which the following can be highlighted:

MAPI films prepared at room temperature present a porous morphology of small grain; however, annealing at 100 °C promotes grain size growth, but the morphology remains porous. Increasing the annealing temperature to 120 °C, the MAPI films show a morphology of compact grains free of pores, but with formation of clusters in some zones. When MAPI films are annealed at a temperature of 140 °C, these present a morphology of compact grains without cluster formation. 

**Figure 12:**
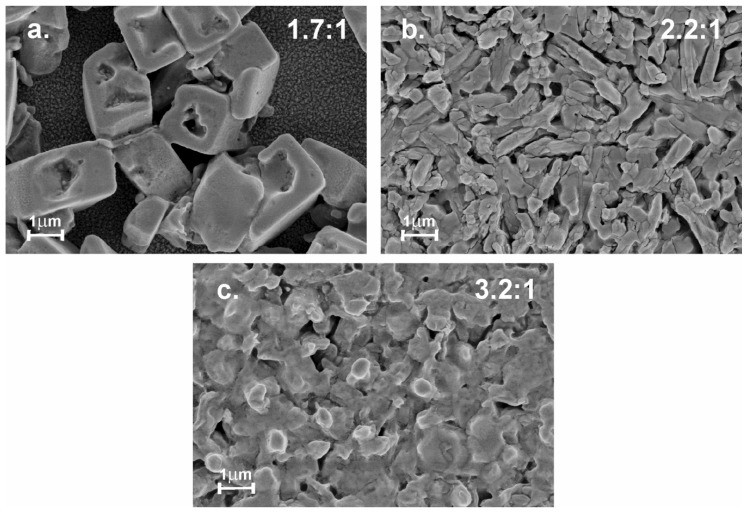
SEM images of FAPI films prepared at room temperature and PbI_2_/FAI thickness ratio of (**a**) 1.7:1, (**b**) 2.2:1 and (**c**) 3.2:1.

Figure 12 shows SEM images corresponding to FAPI films prepared at room temperature varying the composition. This study reveals that samples prepared with an excess of FAI present formation of quasi-cubic grains surrounded by areas of low coverage. 

Based on the results of XRD that indicate that the samples deposited with excess of FAI present formation of the phase FAI (see Figure 5), can be concluded that this morphology is induced by the presence of FAI, that not react with the PbI_2_. On the other hand, samples prepared with excess PbI_2_ exhibit a morphology characterized by formation of compact clusters of micrometric size surrounded by grains of smaller size. The samples of FAPI prepared with chemical composition corresponding to a PbI_2_/MAI thickness ratio of 2.2:1, exhibit a morphology constituted by structures of compact grain of elongated form whose length is in the order of the microns.

### 3.4. Preliminary Results of MAPI and FAPI-Based Solar Cells 

**Figure 13:**
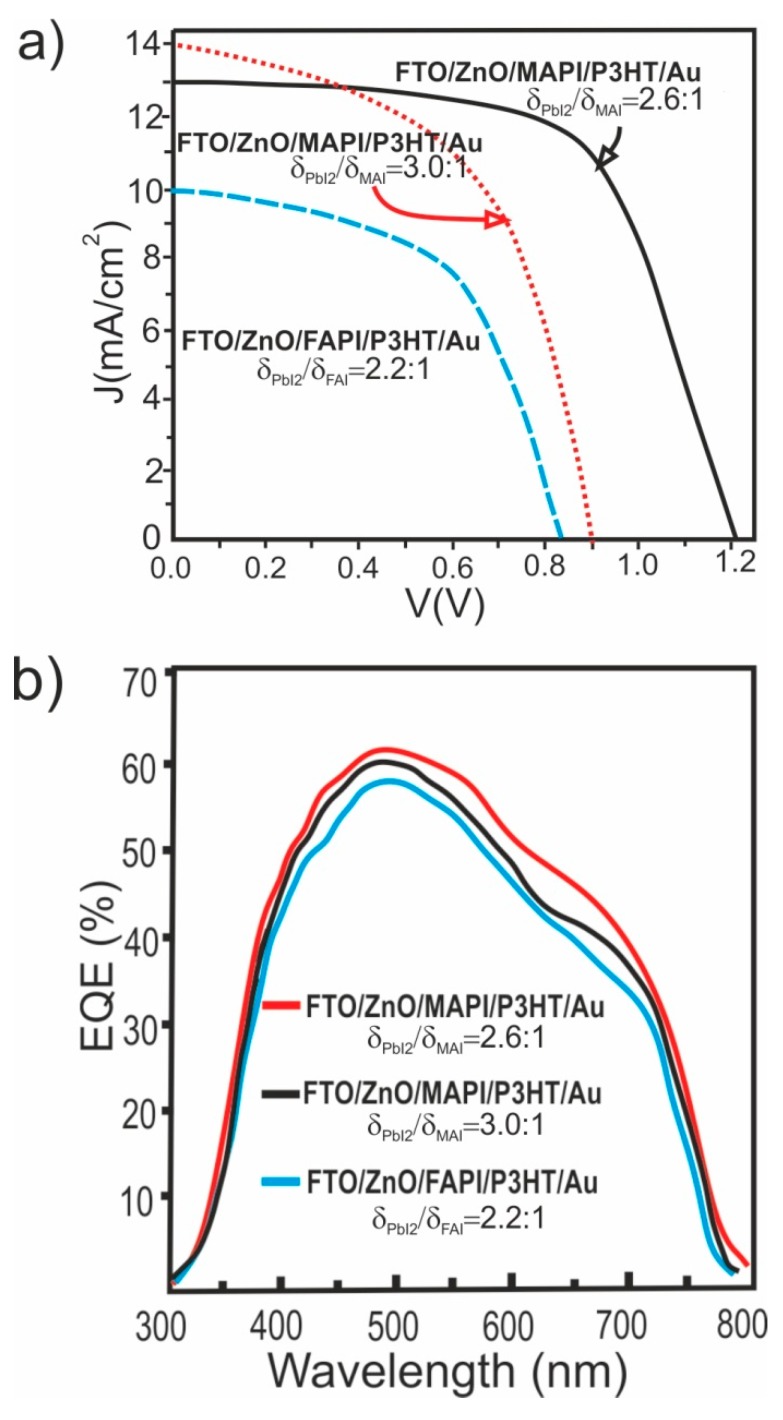
(**a**) J–V curve and (**b**) external quantum efficiency, of the best solar cell fabricated with structure FTO/ZnO/MAPI/P3HT/Au and FTO/ZnO/FAPI/P3HT/Au. $\delta_{PbI2}/\delta_{MAI}$: PbI_2_/MAI thickness ratio and $\delta_{PbI2}/\delta_{FAI}$: PbI_2_/FAI thickness ratio.

With the purpose of testing the applicability of the MAPI and FAPI films grown in this work, as active layer in photovoltaic devices, solar cells with structure FTO/ZnO/MAPI(FAPI)/P3HT/Au were fabricated; the J-V curves were measured under air mass 1.5 (AM 1.5) irradiance (100 mW/cm^2^). Typically, the cells were fabricated using a 150 nm thick electron transport layer (ETL) of ZnO deposited by radio frequency (RF) sputtering, a 300 nm thick MAPI (or FAPI) film deposited by sequential evaporation, a 60 nm thick hole transport layer (HTL) of Poly(3-hexylthiophene) (P3HT) deposited by spin coating and a 0.5 μm thick Au layer used as anode and deposited by RF sputtering. In Figure 13 are displayed the J-V curves and external quantum efficiency (EQE) spectra of the best solar cells that were fabricated using MAPI and FAPI films as the active layer; in Table 3 are listed values of the corresponding performance parameters (short-circuit current density *Jsc*, open-circuit voltage *V_OC_*, fill factor *FF* and efficiency *η*); the effect of the perovskite active layer thickness on the performance parameter are also reported in Table 2.

From Figure 13a it can be seen that the low efficiency of the most solar cells we have fabricated is mainly caused by poor *FF* and low short circuit current (*Jsc*). The low value of *FF* is in part caused by the high values of the device series resistance. The *FF* also depends on the diode quality factor which is affected by recombination through trap centers inside the depletion region [38], so that additional losses of *FF* could be attributed to recombination in the depletion region. The small *Jsc* value of the cells was analyzed through the behavior of the external quantum efficiency (EQE) shown in Figure 13b. The blue part of the EQE shows a pronounced increase associated to the high transparency of the FTO/ZnO/ bilayer; however the EQE decreases at long wavelength; this behavior may be attributed to a reduced minority carrier collection length in the device, apparently due to a poor charge transfer in the interfaces ZnO/MAPI and P3HT/MAPI. High loss of photocurrent by recombination in states of interfaces may be another factor that contributes to the losses of the short circuit current.

The data reported in Table 3 indicate that thickness of the perovskite active layer significantly affects the short circuit current and therefore the device efficiency; the largest short circuit current was obtained with cells fabricated using an active layer of 300 nm thick; nevertheless, when this thickness increases the current decreases strongly. This behavior could be attributed to a short diffusion length of carriers of the perovskite active layer, resulting in a deterioration of the electric transport by increasing the thickness of the active layer. It was also found that the highest efficiencies were obtained with cells manufactured using a MAPI layer with composition corresponding to a PbI_2_/MAI thickness ratio of 2.6:1. This result could be attributed to the fact that MAPI layers prepared under these conditions do not have secondary phases and present better morphology and better crystalline microstructure than FAPI and MAPI prepared under 3:1 PbI_2_/MAI thickness ratio.

Within the framework of this work we were able to manufacture cells with efficiencies of 9.4%, a result that we consider promising, considering that we have used ZnO and P3HT as ETL and HTL layers and that we have not done yet a study to optimize the performance of these layers.

## 4. Conclusions

In this work we were able to grow thin films of MAPI and FAPI with adequate properties and high reproducibility by sequential evaporation of their precursors (lead iodide and methylammonium iodide/formamidinium iodide). This achievement was obtained through the use of a deposition methodology in two stages, for which an electronic system based on virtual instrumentation was designed and implemented; the system allows in a first stage to heat the crucibles through a constant temperature ramp followed by a second phase where the sample is deposited controlling the deposition rate of the precursors.

By correlating characterization results made through measurements of transmittance, XRD, and SEM, with a study of synthesis parameters, we have found that the properties of the MAPI and FAPI films are affected by most of the synthesis parameters: however the chemical composition and post deposition annealing temperature are the parameters that most critically affect them. It was found that single phase MAPI films can be achieved evaporating sequentially PbI_2_ and MAI at room temperature using a PbI_2_/MAI thickness ratio of 2.6 to 1 (molar ratio of approximately 1/1). Samples prepared evaporating excess of PbI_2_ present a mixture of the MAPI and PbI_2_ phases; however, the presence of PbI_2_ can be eliminated by post deposition annealing at 140 °C.

We found that MAPI films grow in general with better optical, structural and morphological properties than FAPI films, which allows obtaining efficiencies significantly higher from devices manufactured using MAPI as active layer that from FAPI films.

The applicability as active layer in hybrid solar cells of the MAPI films we have grown by sequential evaporation of precursor in this work was demonstrated and efficiencies of 9.4% were achieved with solar cells fabricated with structure FTO/ZnO/MAPI/P3HT/Au without having even done a study to optimize the performance of the HTL and ETL layers. 

## Figures and Tables

**Table 1:** List of studied deposition parameters of MAPI and FAPI films and corresponding variation range. MAI: methylammonium iodide, FAI: formamidinium iodide.

Studied Parameter	Range of Variation
PbI_2_/MAI thickness ratio	1.5:1–4.5:1
PbI_2_/FAI thickness ratio	1.5:1–3.2:1
Annealing temperature (°C)	100–140 °C
Annealing time (min)	10–20 min

**Table 2:** Influence of the PbI_2_/MAI (and PbI_2_/FAI) thickness ratio and post deposition annealing temperature on the crystallite size D, grain size, and micro-strain Ɛ of MAPI and FAPI films.

Sample	PbI_2_/MAI and PbI_2_/FAI Ratio	Annealing Temp. (°C)	Annealing Time (min)	M. Scherrer	M. Willamsom–Hall		SEM
				D (nm)	D (nm)	ε × 10^−5^	Grain Size (nm)
MAPI	2.6:1	-	-	158.86	153.92	1.85	492.32
	3:1	140	20	145.81	131.21	2.01	493.23
FAPI	2.2:1	-	-	128.36	112.23	2.34	458.12

**Table 3:** Influence of the thickness and composition of the active layer of perovskita on the performance parameters (*Jsc*, *V_OC_*, *FF* and *η*) of solar cells fabricated with structure FTO/ZnO/Perovskita/P3HT/Au.

Cell Structure	MAI (FAI)/PbI_2_ Thickness Ratio	Perovskite Thickness (nm)	Performance Parameters			
			*Jsc* (mA/cm^2^)	*Voc* (V)	*FF* (%)	*η* (%)
FTO/ZnO/MAPI/P3HT/Au	2.6:1	300	13.2	1.22	0.60	9.4
		400	10.2	0.91	0.47	4.5
		600	8.8	0.92	0.41	3.3
FTO/ZnO/MAPI/P3HT/Au	3:1, (annealed at 140 °C)	300	14	0.9	0.53	6.8
		400	10.7	1.32	0.43	6.1
		600	8.7	1.32	0.36	4.1
FTO/ZnO/FAPI/P3HT/Au	2.2:1	300	10	0.83	0.53	4.4
		400	9.1	0.81	0.47	3.4
		600	7.8	0.87	0.41	2.8
